# Seeking Mental Health Support Among College Students in Video-Based Social Media: Content and Statistical Analysis of YouTube Videos

**DOI:** 10.2196/31944

**Published:** 2021-11-11

**Authors:** Bogeum Choi, Heejun Kim, Jina Huh-Yoo

**Affiliations:** 1 School of Information and Library Science University of North Carolina at Chapel Hill Chapel Hill, NC United States; 2 Department of Information Science University of North Texas Denton, TX United States; 3 College of Computing and Informatics Drexel University Philadelphia, PA United States

**Keywords:** mental health, college student, social media, YouTube, help-seeking, experiential knowledge, video types, content analysis, time distribution analysis, depression, anxiety, student, knowledge, stigma, strategy, engagement

## Abstract

**Background:**

Mental health is a highly stigmatized disease, especially for young people. Due to its free, accessible format, college students increasingly use video-based social media for many aspects of information needs, including how-to tips, career, or health-related needs. The accessibility of video-based social media brings potential in supporting stigmatized contexts, such as college students’ mental health. Understanding which kinds of videos about college students’ mental health have increased viewer engagement will help build a foundation for exploring this potential. Little research has been done to identify video types systematically, how they have changed over time, and their associations on viewer engagement both short term and long term.

**Objective:**

This study aims to identify strategies for using video-based social media to combat stigmatized diseases, such as mental health, among college students. We identify who, with what perspective, purpose, and content, makes up the videos available on social media (ie, YouTube) about college students’ mental health and how these factors associate with viewer engagement. We then identify effective strategies for designing video-based social media content for supporting college students’ mental health.

**Methods:**

We performed inductive content analysis to identify different types of YouTube videos concerning college students’ mental health (N=452) according to video attributes, including poster, perspective, and purpose. Time analysis showed how video types have changed over time. Fisher’s exact test was used to examine the relationships between video attributes. The Mann-Whitney *U* test was used to test the association between video types and viewer engagement. Lastly, we investigated the difference in viewer engagement across time between two major types of videos (ie, individuals’ storytelling and organization’s informational videos).

**Results:**

Time trend analysis showed a notable increase in the number of (1) videos by individuals, (2) videos that represent students’ perspectives, and (3) videos that share stories and experiential knowledge over the recent years. Fisher’s exact test found all video attributes (ie, poster, perspective, and purpose) are significantly correlated with each other. In addition, the Mann-Whitney *U* test found that poster (individual vs organization) and purpose (storytelling vs sharing information) type has a significant association with viewer engagement (*P*<.001). Lastly, individuals’ storytelling videos had a greater engagement in the short term and the long term.

**Conclusions:**

The study shows that YouTube videos on college students’ mental health can be well differentiated by the types of posters and the purpose of the videos. Taken together, the videos where individuals share their personal stories, as well as experiential knowledge (ie, tips and advice), engaged more viewers in both the short term and long term. Individuals’ videos on YouTube showed the potential to support college students' mental health in unique ways, such as providing social support, validating experience, and sharing the positive experience of help-seeking.

## Introduction

### Preface

Postsecondary education represents a peak onset period for mental disorders [1]. Studies showed mental health disorders affect 12%-46% of all university students at any given year [2-4]. However, due to stigma, embarrassment, and preference for self-reliance, only 1 in 5 receives minimally adequate treatment [3]. On the other hand, young adults look for social support and encouragement from others as aids for the help-seeking process [5].

Studies showed college student communities rely heavily on YouTube for both academic and entertainment purposes [6,7]. YouTube, the most popular video-sharing website and the second most popular social media website after Facebook, had 2 billion users as of 2020. A new video is uploaded every minute, and, on average, a user spends at least 15 minutes a day on the site [8,9]. As a result, researchers have been increasingly examining YouTube as a source of health information, including descriptions of various health conditions such as rheumatoid arthritis, weight loss, infertility, anxiety, acute myocardial infarction, and attention deficit disorder [10]. In these studies, the analysis focused on the validity of shared content as factual information about the diseases, prognosis, and symptoms. While misinformation is a significant challenge in social media, one of the benefits of social media is peer support and the personal experiences of others that may be difficult to access otherwise, mainly due to the stigma around sharing about one’s mental illness [11,12].

Studies showed videos containing personal narratives and experiential knowledge are associated with higher viewer engagement and are preferred sources for seeking mental health information [13]. However, one thing that remains unclear about this type of video is their growth rate pattern [14], which is a critical measure for engagement and can be predictive of popularity. No studies of YouTube videos on health have examined growth patterns and what it means to generate constructive and engaging videos that serve as long-lived resources for the target audience. Despite the imminent need, studies have underexplored how video-based social media, such as YouTube, provide a supportive environment for stigmatized diseases such as mental health among college students.

This work investigates how social media provides an environment in which individuals who are susceptible to stigma can benefit from others’ personal narratives and experiential information. To address this goal, we first conducted a systematic search of YouTube videos. We then performed a qualitative content analysis to identify key categories for video attributes such as poster, perspective, and purpose. Then we looked at a distribution of the video attributes over time. Finally, using the categories developed from the content analysis, we performed statistical analyses (ie, Fisher’s exact test and Mann-Whitney *U* test) to investigate (1) the relationships among the video attributes and (2) the association between video attributes and viewer engagement measures such as views, likes, dislikes, and comments.

The findings inform our understanding of the videos published about college students’ mental health, such as what types of videos are published on college students’ mental health, which has become more common over time, and how viewers are engaging them. This finding is essential for informing future work and provides insights for improving some videos for greater engagement.

### Background

College students’ mental health has posed a significant concern in higher education. A WHO (World Health Organization) survey in 19 colleges across 8 countries showed nearly 35% (4894/13984) of college student interviewees met DSM-IV criteria for at least one mental disorder in lifetime and 31% (4335/13984) in the last 12 months, where anxiety and mood disorders were the most prevalent [15]. Other studies have reported similar or even higher prevalence rates of mental disorders among college students [1,16-18]. However, mental health service utilization among this population can be low. A survey study showed, among students with mental disorders, only 36% received any treatment in the past year [19]. The barriers to receiving the treatment include stigma, embarrassment, and preference for self-reliance [5]. On the other hand, students are more open to receiving help when the help is provided through social support and encouragement from peers [20,21]. Due to these reasons, college students are more likely to search for informal alternatives, such as going online [22,23].

Peer support exchanged through social media has received much attention in the research community. By sharing personal experiences and providing emotional support among those with a similar background, researchers examined how internet-based peer support through social media (eg, community forum and YouTube) can positively impact behavior change and health outcomes [24]. Peer engagement and social support can facilitate college students’ help-seeking for mental health issues. Peer support on social media, combined with college students’ heavy use of YouTube [6,7], presents the potential of YouTube videos to encourage college students to acknowledge their mental health issues and seek and receive help. However, there is a lack of investigation on video content, genre, and viewer engagement, especially concerning engagement over time. Therefore, it is critical to investigate how YouTube videos can support this population (ie, what kinds of videos are available related to college students’ mental health and how the viewers have engaged these videos over time). Understanding the content and the types of videos that effectively engage people will help identify and evaluate ways to bring support mechanisms through nonclinical means of communication such as social media.

We conducted a content analysis and statistical analysis of YouTube videos related to college students' mental health and their engagement metrics to address this gap. In addition, we discuss implications for how organizations, groups, and individuals can use video-based social media platforms to support stigmatized conditions that can benefit from peer support.

### Research Question

RQ1: What types of videos are available on YouTube regarding college students’ mental health?

We investigate the video types available on YouTube regarding college students’ mental health by identifying the following attributes of a video: poster, perspective, and purpose. We also examine how the distribution of the identified categories for each video attribute changes over time and if there is a systematic relationship between the video attributes.

RQ2: Do video types have associations with viewer engagement?

We investigate whether the types of video, as defined by video attributes, have any associations with viewer engagement measured by the number of views, likes, dislikes, and comments. We further examine if a video type (ie, individual’s storytelling video vs organization’s informational video) has an association with long-term viewer engagement.

## Methods

### Data Collection

We conducted a keyword search of YouTube videos concerning the mental health of college students. We formulated search keywords based on three topical categories: illness, population, and context. For the illness-related keyword, we selected 4 top symptoms of college students receiving mental health services from the 2019 Center for Collegiate Mental Health Annual Report, including depression, anxiety, social anxiety, and academic distress. Based on Consumer Health Vocabulary, we changed these into more commonly used terms, “depression,” “anxiety,” and “stress” [25]. We also included an overarching term, “mental health,” in this category. For the population-related keyword, we used “college student(s)” and other terms including “university student(s)” and “campus” after reviewing related search terms that YouTube suggested with “college student(s).” We extracted context-related keywords from the initial search results retrieved by illness and population keywords. The goal was to reflect a variety of contexts in which the videos can be sought and used by college students. We then combined the terms from each category (illness, population, and context) and used it as a search query (see Textbox 1).

To alleviate biases from Google’s personalization algorithms, we developed a Python (version 3.8.5; Python Software Foundation) script that drives an Incognito Chrome browser and enables anonymous searches from YouTube. The script ran all the possible combinations of keywords as a query and parsed video IDs from retrieved videos. These IDs were then passed over to the YouTube API to collect metadata (eg, video description, publication date, views, likes, dislikes, and comments) associated with each video.

**Categories and search keywords.**

**Illness:** Depression, anxiety, stress, mental health

**Population:** College student, university student, campus

**Context:** How to, life

**Examples of search query:** Depression college student how to, anxiety university student how to, mental health campus life, etc

One word from each category was used at a time to construct a query.

The search was conducted between May 28, 2020, and May 30, 2020, which resulted in 1930 videos. After removing duplicates, 860 unique videos were obtained. Next, 2 authors screened the video's title and description based on the exclusion criteria (ie, the link is no longer active or not in English) and the inclusion criteria (ie, the video is about college students and mental health). This screening process yielded 452 videos. The 408 videos eliminated during the screening include students preparing for college rather than students enrolled in college or those about general health, not mental health.

### Data Analysis

#### Content Analysis

To address RQ1, we conducted an iterative, inductive content analysis [26]. We identified and categorized relevant information on 3 specific video attributes: (1) poster, (2) perspective, and (3) purpose. The analysis was conducted using the title, written description, and the content of the video. The analysis was led by 1 coder (first author), while the whole team of 4 researchers iteratively discussed the codes and reached a consensus on the most salient code names and descriptions from the data. The coder then revisited the originally coded data and recoded those data using the revised coding scheme developed as a team. We iterated this process until the team agreed upon a final set of code names and definitions. Then we identified categories of related themes across the codes. These categories will be presented as findings for each video attribute.

#### Statistical Analysis

First, we applied time distribution analysis to demonstrate how the distribution of categories of each video attribute changes over time. We plotted the occurrences of the categories of video attributes by year. Additionally, we performed Fisher’s exact test to investigate the relationship between video attributes. We used Fisher’s exact text instead of the chi-square test because our data had categories with a low-frequency value, and using the chi-square test with low cell counts (ie, less than 5) could lead to increased type I errors.

To address RQ2, we first performed the Mann-Whitney *U* test to examine the association between video attributes and viewer engagement scores (ie, views, comments, likes, and dislikes). The Mann-Whitney *U* test, also known as Wilcoxon rank-sum test, can test differences between 2 groups when the data is not normally distributed. Specifically, we examined the following attributes: poster (individual vs organization), perspective (student vs domain expert), purpose (storytelling vs sharing information), and poster combined with the purpose (individual + storytelling vs organization + sharing information). We compared these specific categories because those were the main, distinct categories that make up the videos on college students’ mental health by the content analysis. The viewer engagement scores were normalized to control the confounding effects of exposure time. In other words, the scores (eg, the number of views) were divided by each video’s exposure time. We used R (version 3.6.2; R Core Team) for all statistical analyses.

Lastly, we demonstrated the association between video attributes (specified above) and long-term viewer engagement. This analysis chose the number of comments from the 4 engagement scores as a dependent variable because we believed it to be a more meaningful measure for viewers’ engagement with a video. Prior work also suggests that commenting reflects a higher engagement level than viewing or liking as it involves expressing one’s opinions or feelings in public [27]. Since we had uneven distribution between the groups to be compared (eg, more videos by individuals than organizations), we randomly selected the same number of videos for each group from those published between 2013 and 2019. Then we compared the number of comments in the years following the publication year (n+0, n+1, n+2…where n is the publication year).

## Results

Below, we discuss the findings in response to the two research questions:

1. RQ1: What types of videos are available on YouTube regarding college students’ mental health?
2. RQ2: Do video types have an association with viewer engagement?

### Finding 1. Video Types on YouTube About College Students’ Mental Health

#### Overview

We first present categories identified for the following video attributes: poster, perspective, and purpose of a video. A relative frequency (the portion of times a category occurred in our data) is presented in parentheses. We then show how the distribution of video types has changed over time based on video attributes. Lastly, we present how the video attributes are correlated with each other.

#### Video Attributes

##### Poster

We identified 5 categories for poster types: (1) individual (55%), (2) media (19%), (3) university (18%), (4) health-related organization (6%), and (5) other organization (2%). Individual was coded when a video was created and published by a single individual or a small group of individuals such as a student group. Many of these individuals were college students who either have experienced or are currently experiencing mental health issues. Media included news outlets, television shows, and professional content production groups (eg, TED). University referred to the university itself and university-affiliated organizations that provide mental health-related services to students (eg, student counseling center).

##### Perspective

We identified 5 mutually inclusive categories for the purpose of the videos. Student’s perspective (74%) was the most often represented in the videos, followed by domain expert (32%), others (17%), and amateur coach (7%). Domain expert included mental health experts or those who have expertise in advising students. Amateur coach refers to people who play a role of an advisor based on their life experience while not having credentials in a traditional sense. For example, a public speaker or a podcast host who offers advice on mental health issues was coded as amateur coach.

##### Purpose

We identified 5 mutually inclusive categories for the purpose of the videos. Sharing experiential knowledge was the most frequently identified (58%), followed by sharing information (42%), sharing stories (39%), promoting help-seeking (23%), and sharing awareness (16%). Sharing experiential knowledge was coded when a person in a video shares what they learned from their first-hand experience of mental health. The most common theme under sharing experiential knowledge was sharing tips and advice. Those tips and advice include “strategies to cope with stress and anxiety” (V304), “how to beat depression in college” (V26), “how to deal with stress, anxiety, and depression” (V31), etc. Experiential knowledge is distinct from factual knowledge in 3 significant ways. First, it is drawn from people’s lived experiences, thus more relatable to someone undergoing similar issues. Second, it is often shared to help, support, or guide others. Third, it has a practical value in that viewers can incorporate such tips and advice into the everyday practice of their lives. For example, a person in V312 stated,

…so, I’m going to share with you guys what worked for me, and hopefully, you guys might be able to take some sort of advice from it. Maybe even adopt it and change it into what works for your life.

Sharing information included videos that deliver factual information about mental health (eg, symptoms, causes and treatments, statistics) within and outside an educational setting.

Sharing stories included videos that share personal mental health stories in a narrative style. Some common themes under sharing stories included offering emotional support and sharing an ordinary day of a college student with a mental health issue. Offering emotional support referred to cases where a person in a video showed emotional support for their viewers while disclosing their personal issues. For example, the poster of V56 said,

I opened up to you guys a bit more than I ever have before; I was hesitant to keep that part in the video but decided to push myself out of my comfort zone because hopefully, it helps someone know they're not alone.

In addition, we saw college students share their ordinary days to demonstrate what it is like to be a college student with a mental health issue. For example, the poster of V303 had a series called “uni vlog,” where he showed how he reacted to and coped with a stressful situation in a vlog format. Another poster (V81) discussed her motivation for vlogging as

You can gain a perspective as to what it is for one kid who goes through this and that […] My main goal is to create a vast database of experiences for people to see so they can learn before they have to make the same mistake.

Promoting help-seeking included videos that conveyed help-seeking messages. Help-seeking included not only getting professional help but also talking to friends or family about mental health problems. For example, a poster of V235 said,

Don't be afraid to ask for help. We need to stop feeling like we are super-human. We are really not.

Posters also encouraged others to seek help by sharing their experience of getting professional help during their mental health journey. For instance, the poster of V59 said,

I refused to go to therapy for many years…but looking back…No. Go to therapy. Talk about your emotions. You have to get it out. You are going to have to process those emotions eventually, so just start trying. Go to therapy as much as possible. I promise, regardless of your personal type, it helps.

Similarly, the poster of V48 said,

If you have depression, or you suspect that you have depression, I’d encourage you to try to reach out to a therapist. Maybe you need to just talk to someone. Believe me, I thought that I would never reach out to get professional help. I thought that was completely out of the question. I'm just telling you, if I can do it, you can do it. Trust me.

#### Video Types Over Time

**Figure 1:**
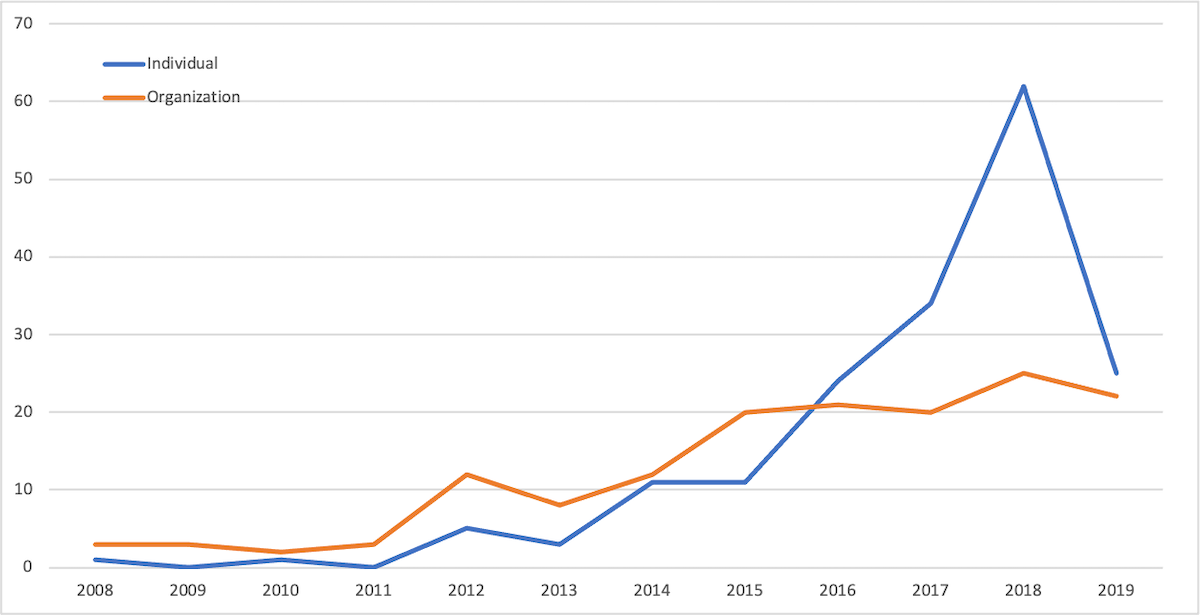
Type of poster by year.

**Figure 2:**
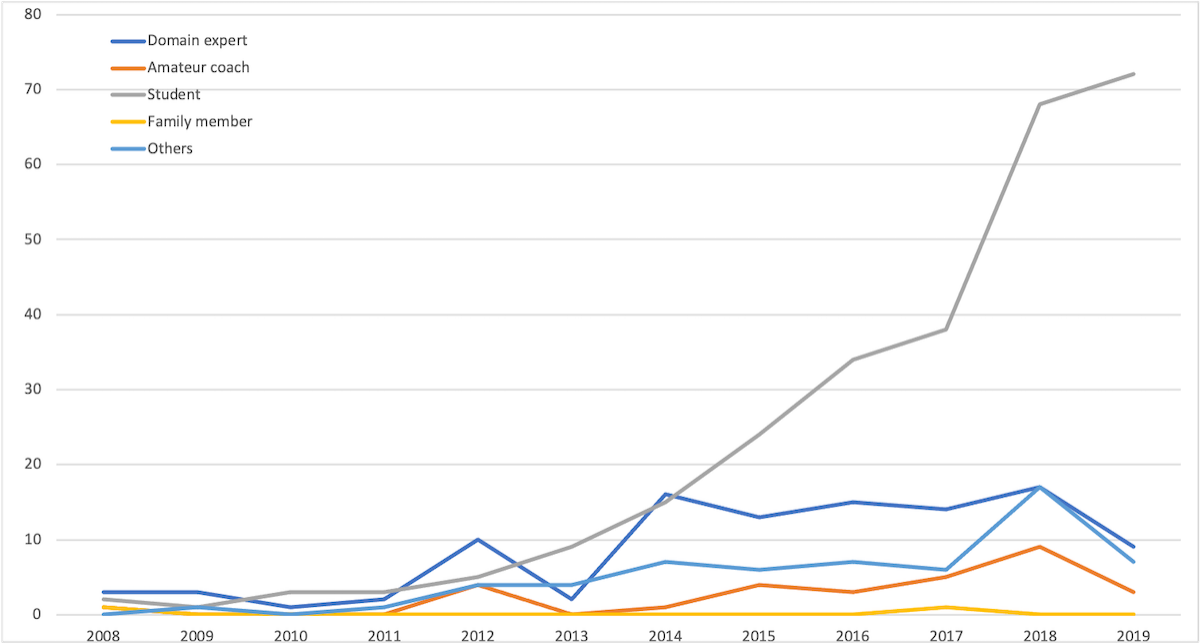
Type of perspective by year.

**Figure 3:**
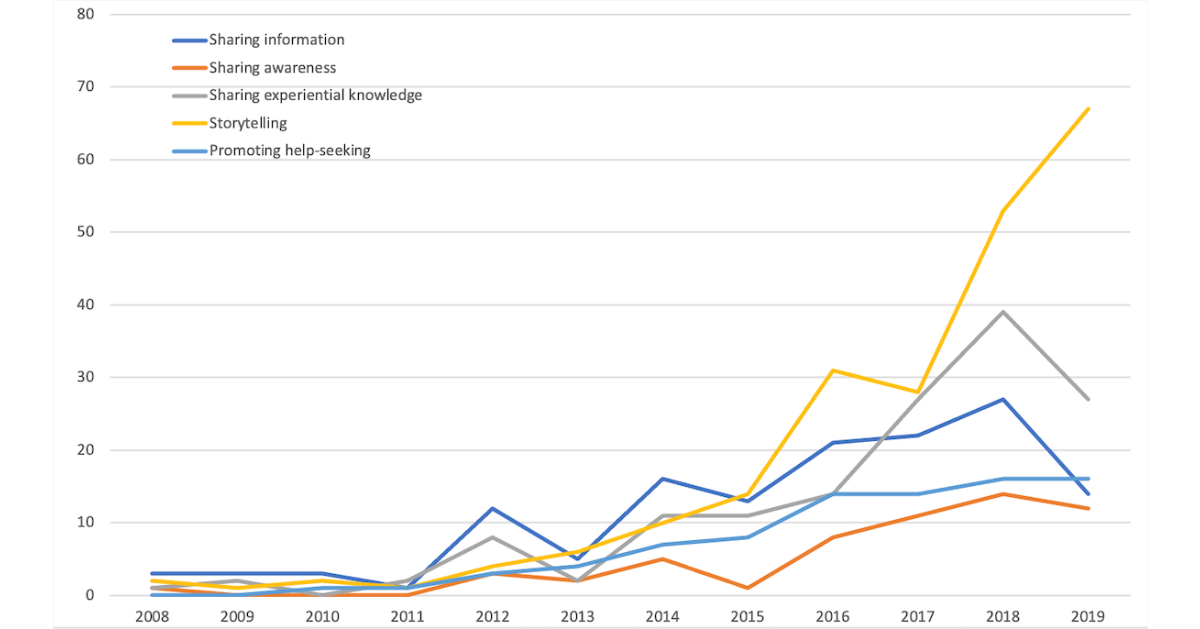
Type of purpose by year.

Based on the identified categories for each video attribute, we looked at how the types of videos posted on YouTube about mental health have changed over time. First, there was a significant increase in videos posted by an individual compared to an organization (Figure 1). Second, the number of videos representing students’ perspectives has increased over the years compared to other perspectives, such as domain experts and amateur coaches (Figure 2). Third, the number of storytelling videos has shown the most significant increase among all-purpose types in the past few years (Figure 3). While educational videos were the most prevalent before 2015, videos sharing personal stories and experiential knowledge have surpassed them since 2016.

#### Relationships Between Video Attributes

We further investigated the relationships between video attributes to see if specific attributes tend to co-occur more. According to Fisher’s exact test, all combinations of our video attributes showed a significant relationship with each other. First, poster type significantly correlated with the perspective type (*P*<.001). As shown in Table 1, individuals’ videos were more likely to include students’ perspectives in their videos, while organizations’ videos were likely to include both student and domain expert perspectives. Poster type and purpose type were also correlated (*P*=.03). As shown in Table 2, individuals’ videos primarily focused on storytelling and sharing experiential knowledge, while organizations’ videos focused on sharing information. Lastly, perspective type significantly correlated with the purpose type (*P*<.001). As shown in Table 3, videos representing students’ perspectives share stories and experiential knowledge, while videos with domain experts’ perspectives share information. Each cell in Tables 1- 3 presents the number of co-occurring attributes and the percentage in parenthesis. As mentioned above, the categories for perspective and purpose were not mutually exclusive, which means a video could be coded as more than one category.

**Table 1:** Number and percentage of poster versus perspective.

Poster		Perspective				
		Student (n=293)	Domain expert (n=116)	Amateur coach (n=31)	Family member (n=2)	Others (n=65)
Individual (n=251), n (%)		189 (47.6)	31 (7.8)	16 (4.0)	0 (0)	15 (3.8)
**Organization (n=258), n (%)**						
	Media (n=123)	47 (11.8)	32 (8.1)	8 (2.0)	2 (0.5)	34 (8.6)
	University (n=94)	44 (11.1)	34 (8.6)	4 (1.0)	0 (0)	12 (3.0)
	Health-related org (n=27)	8 (2.0)	16 (4.0)	2 (0.5)	0 (0)	1 (0.3)
	Other org (n=14)	5 (1.3)	3 (0.8)	1 (0.3)	0 (0)	3 (0.8)

**Table 2:** Number and percentage of poster versus purpose.

Poster			Purpose							
			Share experiential knowledge (n=158)	Share information (n=155)		Share stories (n=237)		Promote help-seeking (n=93)		Share awareness (n=64)
Individual (n=393), n (%)			107 (27.0)	52 (13.1)		165 (41.6)		39 (9.8)		30 (7.6)
**Organization (n=314), n (%)**										
	Media (n=138)	19 (4.8)		52 (13.1)	36 (9.1)		15 (3.8)		16 (4.0)	
	University (n=126)	20 (5.0)		31 (7.8)	29 (7.3)		33 (8.3)		13 (3.3)	
	Health-related org (n=38)	8 (2.0)		17 (4.3)	5 (1.3)		4 (1.0)		4 (1.0)	
	Other org (n=12)	4 (1)		3 (0.8)	2 (0.5)		2 (0.5)		1 (0.3)	

**Table 3:** Number and percentage of perspective versus purpose.

Perspective	Purpose, n (%)				
	Share experiential knowledge (n=190)	Share information (n=248)	Share stories (n=283)	Promote help-seeking (n=122)	Share awareness (n=96)
Student (n=539)	115 (12.2)	77 (8.2)	227 (24.2)	68 (7.2)	52 (5.5)
Domain expert (n=232)	39 (4.2)	97 (10.3)	32 (3.4)	35 (3.7)	29 (3.1)
Amateur coach (n=51)	23 (2.4)	13 (1.4)	7 (0.7)	7 (0.7)	1 (0.1)
Family member (n=6)	0 (0)	2 (0.2)	2 (0.2)	0 (0)	2 (0.2)
Other (n=111)	13 (1.4)	59 (6.3)	15 (1.6)	12 (1.3)	12 (1.3)

### Finding 2. The Association of Video Type With Viewer Engagement

This section presents the association between video types and viewer engagement measured by the number of views, likes, dislikes, and comments. Content analysis informed us that the 2 most distinct types of YouTube videos regarding college students’ mental health could be characterized by the poster and purpose attributes. Thus, we used poster, purpose, and a combination of those two attributes to define video types to compare.

#### The Association Between Video Type and Viewer Engagement

According to the Mann-Whitney *U* test, type of poster, purpose, and poster combined with purpose had significant differences on all measures of viewership engagement (Table 4). Specifically, individuals’ videos (vs organizations’ videos) and storytelling videos (vs informational videos) demonstrated significantly higher viewer engagement than their counterparts. Not surprisingly, individuals’ storytelling videos also demonstrated higher viewer engagement than organizations’ information videos.

**Table 4:** Viewer engagement by video types.

Compared video types		Engagement scores			
		View count	Like count	Dislike count	Comment count
**Poster type (individual vs organization) **					
	Individual, median	3.120	0.096	0.001	0.020
	Organization, median	1.011	0.008	0.000	0.000
	Mann-Whitney *U*	14303.5	10374.0	14214.5	9449.5
	*P* value	<.001	<.001	<.001	<.001
**Purpose type (sharing stories vs sharing information)**					
	Storytelling, median	2.790	0.061	0.001	0.011
	Sharing information, median	0.681	0.005	0.000	0.000
	Mann-Whitney *U*	12853.0	10207.5	14174.0	10563.0
	*P* value	<.001	<.001	<.001	<.001
**Poster + purpose (individual + sharing stories vs organization + sharing information) **					
	Individual + sharing, median	3.502	0.131	0.002	0.030
	Organization + sharing information, median	0.744	0.005	0.000	0.000
	Mann-Whitney *U*	5446.0	3482.0	5943.5	3257.5
	*P* value	<.001	<.001	<.001	<.001

#### The Association Between Video Type and Viewer Engagement Over Time

**Figure 4:**
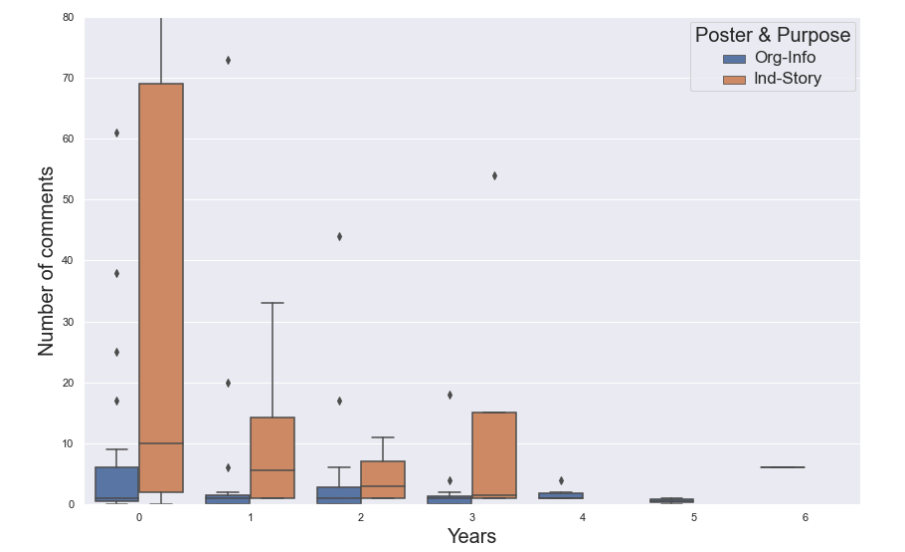
Number of comments by poster-purpose type (individual-sharing stories versus organization-sharing information).

Figure 4 shows how the association between video type (ie, individual’s storytelling vs organization’s informational videos) and viewer engagement varies over time. Individuals’ storytelling videos had a significantly higher engagement at the time of posting and continued to attract more engagements than organizations’ informational videos after some time since posting.

## Discussion

### Principal Findings

This study sought to understand the videos available on YouTube regarding college students’ mental health (RQ1) and the relationships between video types and viewer engagement scores (RQ2).

We found an increasing number of individuals posting videos on YouTube to share their experiential knowledge and personal stories related to mental health. The trend has gotten more robust over time. Individuals’ storytelling videos were associated with greater engagement (ie, views, likes, dislikes, and comments) than organizations’ informational videos. Moreover, individuals’ storytelling videos seem to continue getting engagement (in terms of the number of comments) sometime after the posting. This finding might indicate that users seeking mental health-related videos about college students on YouTube may want to see and hear from those in a similar situation talking about their first-hand experience. Indeed, previous studies found that people use social media platforms for peer support [28,29]. In our data, individuals’ storytelling videos seem to meet such needs among college students, especially considering that many of the storytelling videos are intended to offer emotional support and share everyday life from the first-person perspective. However, we need a careful examination to know the causal relationship between video types and viewer engagement scores. 

Personal stories shared in individuals’ videos can have other specific benefits. Many of these videos included individuals’ recovery narratives. Previous research noted positive outcomes of patients receiving recovery narratives, including connectedness, hope, stigma reduction [30], and validation of experience [31]. Lack of a benchmark can hinder people from recognizing their symptoms, resulting in a barrier to help-seeking [32]. In this regard, the first-hand experience shared in individuals’ videos on YouTube can serve as a reference point while providing validation to college student viewers with mental health issues. This approach can be especially beneficial to individuals whose cultural background may be more prone to the stigma around decreased mental health than others, such as African-American [33] or Asian populations [34]. This benefit will be sustainable if YouTube, as a platform, provides culturally sensitive, accessible information and experience sharing in those populations.

We also found that individuals’ videos frequently share experiential knowledge, which referred to experience-based tips and advice. This content includes activities and mindsets that one can incorporate into their daily lives to improve mental health or better cope with a specific mental health issue (eg, social anxiety). Previous research suggests that people choose to use social media platforms instead of search engines to seek other people’s recommendations and advice on how to manage mental health in particular [35,36] and learn from it, especially among young people [37]. The growing prevalence of videos that share experiential knowledge suggests a potential benefit of giving college students viewers access to other people’s lived experiences.

It is worth noting that the portion of individuals’ videos that promote help-seeking (39/397, 9.8%) was comparable with that of organizations’ videos (54/397, 13.5%) in this study. Previous research found that patients’ perceived treatment outcome is prone to social influence from other patients’ shared experiences [38]. Moreover, our study shows that individuals’ videos get more engagement than organizations’. This finding suggests that individuals’ videos promoting help-seeking while sharing their mental health journey could influence viewers to have a positive attitude towards help-seeking, including formal mental health clinics and services.

Our findings suggest that organizations (eg, universities, health-related organizations) may benefit from incorporating the communication styles used in individuals’ videos when reaching college students about mental health on a large scale. For instance, universities can promote their mental health service by including individual students’ anecdotes about their experience using the service to improve their mental health. Some organizations’ videos included clips of students being interviewed in our data, but many of them were either too short or formal. Organizations may gain greater engagement and increase help-seeking among college student viewers by incorporating more informal personal narratives in delivering mental health-related content.

### Strengths and Limitations

Our study has several strengths and limitations. We provided multiple pieces of evidence that allude to the growing popularity and engagement of individuals’ storytelling videos, including the changing prevalence of videos with different attributes and the association between video types and viewer engagement. As far as we know, no previous research has investigated viewer engagement with videos on the topics of college students’ mental health over time. We included this analysis to see if the trend of individuals’ storytelling videos getting more engagement holds over time. We then subsequently discussed how those individuals’ videos could support college students with mental health issues in more detail based on the qualitative analysis conducted to identify categories of video attributes. We used quantitative measures such as the number of views, likes, dislikes, and comments. Although these measures available from YouTube allow us to gauge the popularity and accessibility of videos, they do not inform if and how videos impact viewers’ mental health. To investigate the actual impact of videos with specific attributes, in our future work, we plan to expand our data collection to include college students’ responses to different types of videos.

### Conclusions

Our study defined different mental health-related videos intended for college students based on video attributes (eg, poster and purpose) and examined engagement with those different video types. Our findings show individuals’ videos that share stories and experiential knowledge have become more popular over time and tend to gain more engagement from viewers for both the short term and long term. Such videos may attract and sustain engagement due to their benefits to college students with mental health issues, including peer support, validating experience, learning from others’ experiences, and encouraging help-seeking. While the quality of health information on social media platforms is debatable, it is essential to consider that there are different types of mental health needs of college students that can be more effectively addressed by individually-generated content on social media such as YouTube. When making interventions targeting help-seeking for mental health issues among college students, incorporating the communication styles used by individuals on social media such as storytelling will be strategic.
